# Magnetic Resonance Image of Neonatal Acute Bilirubin Encephalopathy: A Diffusion Kurtosis Imaging Study

**DOI:** 10.3389/fneur.2021.645534

**Published:** 2021-08-25

**Authors:** Hongyi Zheng, Jiefen Lin, Qihuan Lin, Wenbin Zheng

**Affiliations:** Department of Radiology, The Second Affiliated Hospital, Medical College of Shantou University, Shantou, China

**Keywords:** diffusion kurtosis imaging, DKI, acute bilirubin encephalopathy, ABE, MRI, hyperbilirubinemia, MK

## Abstract

**Background and Objective:** The abnormal T1-weighted imaging of MRI can be used to characterize neonatal acute bilirubin encephalopathy (ABE) in newborns, but has limited use in evaluating the severity and prognosis of ABE. This study aims to assess the value of diffusion kurtosis imaging (DKI) in detecting ABE and understanding its pathogenesis.

**Method:** Seventy-six newborns with hyperbilirubinemia were grouped into three groups (mild group, moderate group, and severe group) based on serum bilirubin levels. All the patients underwent conventional MRI and DKI serial, as well as 40 healthy full-term infants (control group). The regions of interest (ROIs) were the bilateral globus pallidus, dorsal thalamus, frontal lobe, auditory radiation, superior temporal gyrus, substantia nigra, hippocampus, putamen, and inferior olivary nucleus. The values of mean diffusivity (MD), axial kurtosis (AK), radial kurtosis (RK), and mean kurtosis (MK), and fractional anisotropy (FA), radial diffusivity (RD), and axis diffusivity (AD) of the ROIs were evaluated. All newborns were followed up and evaluated using the Denver Development Screening Test (DDST). According to the follow-up results, the patients were divided into the normal group, the suspicious abnormal group, and the abnormal group.

**Result:** Compared with the control group, significant differences were observed with the increased MK of dorsal thalamus, AD of globus pallidus in the moderate group, and increased RD, MK, AK, and RK value of globus pallidus, dorsal thalamus, auditory radiation, superior temporal gyrus, and hippocampus in the severe group. The peak value of total serum bilirubin was moderately correlated with the MK of globus pallidus, dorsal thalamus, and auditory radiation and was positively correlated with the other kurtosis value. Out of 76 patients, 40 finished the DDST, and only 9 patients showed an abnormality. Compared with the normal group, the AK value of inferior olivary nucleus showed significant differences (*p* < 0.05) in the suspicious abnormal group, and the MK of globus pallidus, temporal gyrus, and auditory radiation; RK of globus pallidus, dorsal thalamus, and auditory radiation; and MD of globus pallidus showed significant differences (*p* < 0.05) in the abnormal group.

**Conclusion:** DKI can reflect the subtle structural changes of neonatal ABE, and MK is a sensitive indicator to indicate the severity of brain damage.

## Introduction

Hyperbilirubinemia is one of the most common neonatal disorders (1). High levels of indirect free bilirubin, which is not bound to albumin in the blood, can impact the central nervous system by crossing the blood–brain barrier and precipitating in the brain cells (2). Acute bilirubin encephalopathy (ABE), which is characterized by a progressive disorder of neural behavior, can result in death or lifelong neurodevelopmental disabilities (3–5). In newborns with acute bilirubin toxicity, symptomatic progression begins with poor feeding and lethargy and progresses to initial hypotonia followed by hypertonia, opisthotonus and retrocollis, seizures, and fever (6, 7). As ABE progresses, there is a high risk of permanent neural damage, although some cases have shown that brain cell damage can be transient and minimal with aggressive treatment (2, 8).

Magnetic resonance imaging (MRI) is the most valuable radiologic examination in the diagnosis of bilirubin encephalopathy. The globus pallidus (GP) and subthalamic nucleus (SN) are the most remarkable lesions, and other lesions of ABE include the selected brainstem nuclei, the CA2–CA3 sectors of the hippocampus, the reticular portion of the substantia nigra, and the dentate, roof nuclei, and Purkinje cells of the cerebellum (9). Symmetrically increased signal intensity on T1-weighted images of the globus pallidus, subthalamic nuclei, and hippocampus is the characteristic MRI findings at the acute stage of bilirubin encephalopathy (10). However, in chronic bilirubin encephalopathy, T2-weighted hyperintense imaging of the globus pallidus and subthalamic nuclei is more accurate than T1-weighted imaging (11, 12).

Diffusion kurtosis imaging (DKI) is a newly emerging MRI modality based on the non-Gaussian diffusion of water in biological systems and the index of kurtosis. With DKI, not only are the conventional diffusion tensor imaging (DTI) parameters derivable but also the complexity and heterogeneity of the microenvironments can be distinguished—indicating its potential as a more sensitive biomarker than DTI to pathophysiological changes in the brain. A scalar index derived from DKI called the mean kurtosis (MK) has been shown to be sensitive to structural changes in both anisotropic tissue, such as white matter (WM), and isotropic tissue, such as gray matter (GM), and therefore may provide information on tissue microarchitecture complementary to that given by fractional anisotropy (FA) and mean diffusivity (MD) (13, 14). Current preliminary studies of DKI in human and rat brain tissue infarction, multiple sclerosis, attention deficit hyperactivity disorder, Parkinson's disease, Alzheimer's disease, gliomas, and other central nervous system diseases have achieved notably favorable results (15–18). To date, there were few studies using DKI to evaluate ABE (19).

This study aimed to evaluate the diagnostic value of DKI for ABE. We hypothesized that the values of DKI changing at the early stage of ABE is very important in the assessment of disease progression and prognosis.

## Materials and Methods

### Subjects

This study comprised 76 newborns (33 girls, 43 boys) aged 1–28 days, diagnosed with bilirubin encephalopathy and hyperbilirubinemia; all of the newborns were referred directly to our neonatal intensive care unit without receiving any prior therapeutic interventions for their hyperbilirubinemia state and underwent MRI scan within 2 days. All children were followed up by telephone after discharge and were evaluated using the Denver Development Screening Test (DDST). A similarly aged group of 40 (17 girls, 23 boys) control subjects were enrolled to study from pediatric, neonatology, and neurology clinic patients who were followed for a different diagnosis outside the central nervous system. This study was conducted with approval from the local Ethics Committee of the hospital (No. 2017-48). We discussed the objective of the study and the advantage of MRI, as well as the disadvantage of the sedative, and made sure that the parents fully understood the process. Written informed consent was obtained from all participants' guardians.

#### Inclusion Criteria

All newborns whose gestational age was ≥37 weeks displayed symptoms of jaundice, hypertonia, poor feeding, and lethargy. All the patients were diagnosed with neonatal bilirubin encephalopathy or hyperbilirubinemia. Infants with a peak serum bilirubin value >221 μmol/L were included in the study.

#### Exclusion Criteria

Newborns with congenital dysplasia of the nervous system, respiratory distress, and neurological damage caused by reasons other than bilirubin encephalopathy were excluded from the study.

#### Grouping

According to peak total serum bilirubin (TSB) levels, the newborns with ABE were divided into three groups: a mild group (221 μmol/L ≤ TSB < 342 μmol/L), a moderate group (342 μmol/L ≤ TSB < 428 μmol/L), and a severe group (TSB ≥ 428 μmol/L).

All newborns with ABE discharged from the hospital were followed up by telephone using the DDST to evaluate their intellectual development and the effects of early treatment and intervention training. According to the follow-up results, the patients were divided into the normal group, the suspicious abnormal group, and the abnormal group.

### MRI Imaging

All newborns underwent cranial conventional MRI and DKI evaluations. The newborns were examined after sedation with oral chloral hydrate 50 mg/kg. Cranial MRI was performed with a 3.0 MR imaging system (Signa; GE Healthcare, Milwaukee, WI, USA) with an 8-channel head coil (HD 8Ch HiRes BRAIN ARRAY, GE Healthcare). Axial and sagittal T1-weighted Fluid-attenuated Inversion Recovery (T1W-FLAIR) imaging (TR: 1,750 ms, TE: 24 ms), axial T2-weighted Fast Spin Echo (T2W-FSE) imaging (TR: 4,600 ms, TE: 105 ms), and axial DWI (TR: 5,200 ms, TE: 75 ms) with slice thickness/spacing 4 mm/0.5 mm, FOV 240 mm × 240 mm were obtained. To measure the individual DKI data, the imaging parameters were set as follows: 29 slices, slice thickness: 3 mm, slice spacing: 1 mm, TR: 6,500 ms, TE: 115 ms, FOV: 240 mm × 240 mm, acquisition time: 4 min 6 s. DKI was applied in 15 encoding diffusion directions at three b values (0, 1,000, and 2,000 s/mm^2^).

### DKI Processing

**Figure 1:**
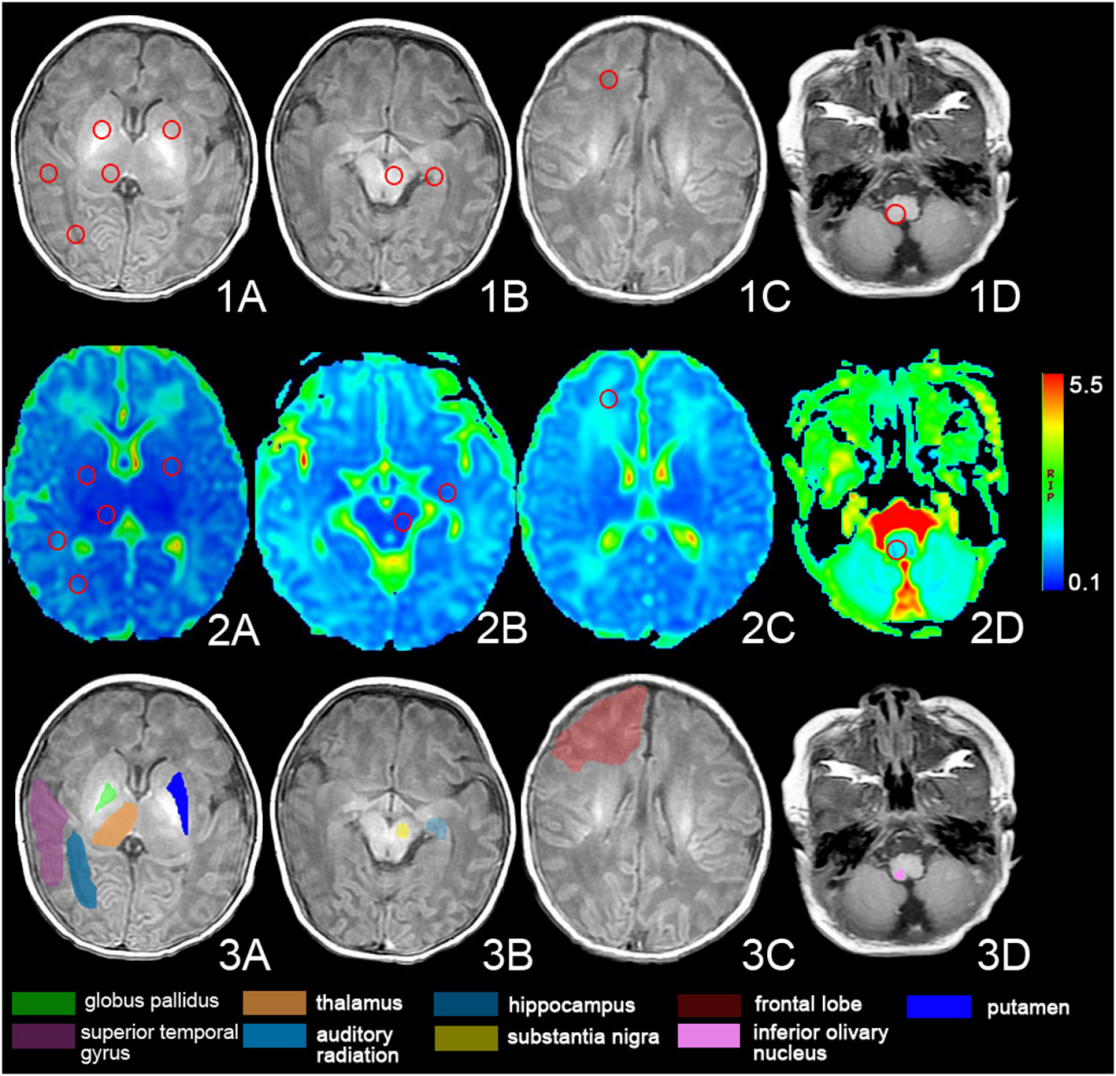
The regions of interest (ROIs) of the DKI. Images show the ROIs of T1-weighted images (1. A–D) and DKI images (2. A–D) and the anatomy of the ROIs (3. A–D). The ROI was set in bilateral globus pallidus, dorsal thalamus, frontal lobe, auditory radiation, superior temporal gyrus, substantia nigra, hippocampus, putamen, and inferior olivary nucleus.

All metrics were obtained using DKI software on the Functool platform of the workstation (Advantage Workstation 4.6) provided by GE Healthcare. The maps of DKI metrics could be generated automatically by the Functool platform of the workstation. The regions of interest (ROI) were set in the bilateral globus pallidus, dorsal thalamus, frontal lobe, auditory radiation, superior temporal gyrus, substantia nigra, hippocampus, putamen, and inferior olivary nucleus (Figure 1). The values of MD and kurtosis (MK, AK, and RK), and AD, RD, and FA of the ROIs were evaluated at the DKI slice, which has the maximum ROI volume. Selected ROIs were placed on non-diffusion-weighted images, with kurtosis maps used for reference. The ROIs, approximately 12 ± 2 mm^2^, were measured three times bilaterally and an average size was calculated to minimize the error value. The images were interpreted independently by two experienced radiologists who were blinded to the neurological manifestations and the results of the analyses.

### Statistical Analysis

Data were analyzed using SPSS 20.0 (SPSS 20.0, IBM, Armonk, NY). The results were presented as means and standard deviations of the DKI parameters in the ROIs in the control and patient groups. Continuous variables of the Apgar scores and the age of the newborns at the moment of the MRI acquisition were expressed as median and interquartile ranges. Categorical variables were reported as whole numbers.

Continuous parameters were checked for normality of distribution using the Shapiro–Wilks test and compared using Kruskal–Wallis *H*-test. A comparison between the case group and the control group was performed by an Independent Samples *t*-test to evaluate the DKI parameters. A chi-square test was used to evaluate the positive rate of T1 hyperintensity of globus pallidus and the positive rate of follow-up result. The value of *p* < 0.05 was considered statistically significant. Pearson's correlation analysis was used to test the relationships between DKI parameters and TBS value. The closer the |*r*| value was to 1, the stronger the correlation between variables.

## Results

### Patient Characteristics

Forty-three patients of the neonatal bilirubin encephalopathy newborns (56.6%) were male and 33 (43.4%) were female. Their average weight was 3.16 ± 0.54 kg, average TSB was 385.8 ± 114.1 μmol/L, and bilirubin levels were in the range of 221.1–860.4 μmol/L. Twenty-three newborns from the control group (57.5%) were male and 17 newborns (42.5%) were female. Their average weight was 2.97 ± 0.73 kg. There was no statistical significance between the case group and the control group in sex and weight (*p* > 0.05). There were also no significant differences in the Apgar scores and the age of the newborns at the moment of the MRI acquisition in different groups (*p* > 0.05) (Table 1). The main cause of jaundice was hemolysis due to ABO incompatibility (32 newborns, 42.1%), and the other causes included G6PD deficiency (14 newborns, 18.4%), intracranial hematoma (6 newborns, 7.9%), septicemia (11 newborns, 14.5%), breast-feeding jaundice (2 newborns, 2.6%) and unknown causes (11 newborns, 14.5%).

**Table 1:** Characteristics of participants.

	**Control**	**Mild group**	**Moderate group**	**Severe group**	***p*-value**
Time, d	6 (2, 11)	6 (4, 10.5)	6 (3.5, 11)	9 (6.25, 10)	0.299
Apgar, 1 min	9 (8, 10)	10 (8.25, 10)	10 (9, 10)	10 (9, 10)	0.482
Apgar, 5 min	10 (9.25, 10)	10 (10, 10)	10 (10, 10)	10 (10, 10)	0.065
Apgar, 10 min	10 (10, 10)	10 (10, 10)	10 (10, 10)	10 (10, 10)	0.888

*Data are expressed as median (interquartile range). Time indicates the age of the infants at the moment of the MRI acquisition; and d, day. Apgar scores of 1, 5, and 10 min were compared among different groups*.

### MRI Findings

#### Conventional MRI

In 48 of 76 newborns (63.2%), there was a bilateral, symmetric, abnormal increased signal intensity in the globus pallidus on T1-weighted imaging, which was not apparent on T2-weighted imaging and diffusion-weighted imaging. According to serum bilirubin levels, the newborns with abnormal MRI were distributed in the mild group (10 newborns), moderate group (18 newborns), and severe group (20 newborns). The high signal positive rate of globus pallidus on MRI T1WI in different groups increases with serum bilirubin and there was a significant difference between the positive rate and serum bilirubin level (*p* < 0.05) (Table 2).

**Table 2:** Comparison of a high signal positive rate of globus pallidus on MRI T1WI in different groups of children (chi-square test, *p* < 0.05).

**Group**	**Total number**	**Positive rate, *n* (%)**
Mild Group	32	10 (31.3%)
Moderate Group	23	18 (78.3%)
Severe Group	21	20 (95.2%)

#### Diffusion Kurtosis Imaging

We compared each patient group according to TSB with the control group, and there were significant differences between groups in the globus pallidus, dorsal thalamus, frontal lobe, auditory radiation, superior temporal gyrus, and hippocampus. In the mild group, the FA values in auditory radiation showed significant differences (*t* = 2.732, *p* < 0.05) compared with the control group. In the moderate group, the MK and RK values of the dorsal thalamus and AD values of the globus pallidus differed from the control group, and the differences were significant [*t*(MK) = 2.265, *t*(RK) = 2.035, *t*(AD) = 2.657, *p* < 0.05]. There were significant differences of DKI parameters in the severe group compared with the control group, including the MK, AK, and RK values of globus pallidus [*t*(MK) = 2.868, *t*(AK) = 2.183, *t*(RK) = 2.665, *p* < 0.05], MK and RK values of dorsal thalamus [*t*(MK) = 2.875, *t*(RK) = 2.216, *p* < 0.05], MK, AK, and RK values of auditory radiation [*t*(MK) = 2.977, *t*(AK) = 3.156, *t*(RK) = 2.457, *p* < 0.05], RD, MK, and AK values of superior temporal gyrus [*t*(RD) = 1.325, *t*(MK) = 2.975, *t*(AK) = 4.306, *p* < 0.05], and MK, RK values of the hippocampus [*t*(MK) = 2.363, *t*(RK) = 2.133, *p* < 0.05] (Table 3). Compared with the mild group, in the moderate group, there were significant differences of DKI parameters including the MK values of globus pallidus (*t* = 2.334, *p* < 0.05), MK and RK values of thalamus [*t*(MK) = 3.074, *t*(RK) = 2.358, *p* < 0.05], MK, RK, and RD values of auditory radiation [*t*(MK) = 2.150, *t*(RK) = 2.337, *t* (RD) = 3.129], MK and RK of superior temporal gyrus [*t*(MK) = 2.388, *t*(RK) = 2.605, *p* < 0.05], and FA and RD of hippocampus [*t*(FA) = 2.953, *t*(RD) = 3.255, *p* < 0.05]; in the severe group, there were significant differences of DKI parameters including the MK, AK, and RK values of globus pallidus [*t*(MK) = 4.372, *t*(AK) = 2.271, *t*(RK) = 2.913, *p* < 0.05], MK, AK, and RK values of thalamus [*t*(MK) = 4.188, *t*(AK) = 2.321, *t*(RK) = 2.986, *p* < 0.05], MK, AK, and RK values of auditory radiation [*t*(MK) = 4.493, *t*(AK) = 2.360, *t*(RK) = 3.366, *p* < 0.05], MK, AK, RK, and FA values of superior temporal gyrus [*t*(MK) = 5.058, *t*(AK) = 3.871, *t*(RK) = 3.094, *t*(FA) = 2.895, *p* < 0.05], MK, AK, and RK of hippocampus [*t*(MK) = 3.385, *t*(AK) = 2.699, *t*(RK) = 2.199, *p* < 0.05], and MK and AK values of putamen [*t*(MK) = 3.175, *t*(AK) = 2.674, *p* < 0.05]. Compared with the moderate group, there were significant differences in DKI parameters in the severe group, including the MK values of globus pallidus [*t*(MK) = 2.252, *p* < 0.05], MK and AK values of auditory radiation [*t*(MK) = 2.755, *t*(AK) = 3.309, *p* < 0.05], MK and AK of superior temporal gyrus [*t*(MK) = 2.967, *t*(AK) = 4.525, *p* < 0.05], and MK values of putamen [*t*(MK) = 2.576, *p* < 0.05].

**Table 3:** Comparison of DKI parameters for each ROI of the control group and case group.

		**Control**	**Mild**	**Moderate**	**Severe**
Globus Pallidus	MK	0.295 ± 0.034	0.293 ± 0.025	0.328 ± 0.013^γ^	0.333 ± 0.041^*^^γ^^β^
	AK	0.309 ± 0.052	0.326 ± 0.046	0.318 ± 0.045	0.356 ± 0.055^*^^γ^
	RK	0.282 ± 0.036	0.285 ± 0.059	0.319 ± 0.018	0.325 ± 0.053^*^^γ^
	FA	0.175 ± 0.021	0.182 ± 0.022	0.182 ± 0.016	0.170 ± 0.025
	MD	1.158 ± 0.068	1.179 ± 0.055	1.174 ± 0.070	1.194 ± 0.178
	AD	1.442 ± 0.102	1.473 ± 0.094	1.558 ± 0.133^*^	1.490 ± 0.239
	RD	1.058 ± 0.060	1.088 ± 0.057	1.046 ± 0.121	1.046 ± 0.173
Thalamus	MK	0.283 ± 0.041	0.262 ± 0.045	0.310 ± 0.035^*^^γ^	0.324 ± 0.052^*^^γ^
	AK	0.335 ± 0.052	0.336 ± 0.065	0.352 ± 0.061^*^	0.367 ± 0.063^γ^
	RK	0.255 ± 0.044	0.254 ± 0.045	0.283 ± 0.051^γ^	0.292 ± 0.069^*^^γ^
	FA	0.160 ± 0.039	0.164 ± 0.022	0.160 ± 0.258	0.154 ± 0.059
	MD	1.119 ± 0.051	1.135 ± 0.073	1.107 ± 0.138	1.109 ± 0.171
	AD	1.295 ± 0.060	1.292 ± 0.058	1.295 ± 0.060	1.327 ± 0.213
	RD	0.988 ± 0.079	1.004 ± 0.055	1.030 ± 0.064	1.002 ± 0.171
Auditory Radiation	MK	0.296 ± 0.061	0.295 ± 0.031	0.319 ± 0.027^γ^	0.342 ± 0.055^*^^γ^^β^
	AK	0.266 ± 0.066	0.283 ± 0.064	0.288 ± 0.031	0.323 ± 0.049^*^^γ^^β^
	RK	0.308 ± 0.084	0.314 ± 0.059	0.338 ± 0.051^γ^	0.372 ± 0.082^*^^γ^
	FA	0.274 ± 0.045	0.306 ± 0.035^*^	0.296 ± 0.037	0.293 ± 0.052
	MD	1.339 ± 0.121	1.375 ± 0.091	1.365 ± 0.099	1.360 ± 0.248
	AD	1.809 ± 0.134	1.796 ± 0.107	1.641 ± 0.151^γ^	1.598 ± 0.134
	RD	1.270 ± 0.114	1.255 ± 0.131	1.304 ± 0.129^γ^	1.258 ± 0.225
Superior Temporal Gyrus	MK	0.276 ± 0.038	0.265 ± 0.023	0.289 ± 0.017^γ^	0.318 ± 0.045^*^^γ^^β^
	AK	0.348 ± 0.042	0.355 ± 0.045	0.358 ± 0.036	0.407 ± 0.046^*^^γ^^β^
	RK	0.246 ± 0.041	0.236 ± 0.031	0.262 ± 0.029^γ^	0.273 ± 0.053^γ^
	FA	0.152 ± 0.026	0.161 ± 0.025	0.146 ± 0.041	0.136 ± 0.031^γ^
	MD	1.329 ± 0.121	1.376 ± 0.096	1.365 ± 0.083	1.360 ± 0.248
	AD	1.594 ± 0.087	1.599 ± 0.108	1.594 ± 0.087	1.645 ± 0.290
	RD	1.257 ± 0.089	1.272 ± 0.102	1.332 ± 0.110	1.336 ± 0.212^*^
Hippocampus	MK	0.379 ± 0.064	0.365 ± 0.032	0.376 ± 0.061	0.422 ± 0.084^*^^γ^
	AK	0.434 ± 0.069	0.419 ± 0.045	0.421 ± 0.051	0.489 ± 0.131^γ^
	RK	0.344 ± 0.084	0.354 ± 0.048	0.362 ± 0.078	0.396 ± 0.086^*^^γ^
	FA	0.149 ± 0.055	0.158 ± 0.021	0.165 ± 0.039^γ^	0.158 ± 0.041
	MD	1.372 ± 0.142	1.334 ± 0.115	1.319 ± 0.168	1.374 ± 0.201
	AD	1.378 ± 0.087	1.404 ± 0.097	1.378 ± 0.087^γ^	1.406 ± 0.239
Putamen	RD	1.055 ± 0.115	1.055 ± 0.056	1.148 ± 0.096^γ^	1.140 ± 0.187
	MK	0.276 ± 0.035	0.268 ± 0.034	0.271 ± 0.031	0.307 ± 0.045^*^^γ^^β^
	AK	0.343 ± 0.046	0.321 ± 0.055	0.336 ± 0.048	0.394 ± 0.118^γ^
	RK	0.245 ± 0.035	0.244 ± 0.050	0.240 ± 0.056	0.267 ± 0.050
	FA	0.150 ± 0.014	0.149 ± 0.020	0.144 ± 0.023	0.144 ± 0.023
	MD	1.127 ± 0.091	1.157 ± 0.051	1.146 ± 0.053	1.150 ± 0.186
	AD	1.343 ± 0.130	1.367 ± 0.056	1.319 ± 0.050	1.297 ± 0.213
	RD	1.129 ± 0.130	1.104 ± 0.075	1.130 ± 0.040	1.081 ± 0.214

*The DKI parameters of each ROI between the two groups were compared with an independent t-test*.

* *p < 0.05 was considered to indicate a statistically significant difference*.

γ *p < 0.05 was considered to indicate a statistically significant difference compared with the mild group*.

β *p < 0.05 was considered to indicate a statistically significant difference compared with the moderate group*.

We also compared the correlation of peak TSB values with DKI parameters of different ROIs. There was a significant positive correlation in the MK, AK, and RK of globus pallidus, MK and RK of dorsal thalamus, MK, AK, and RK of auditory radiation, MK, AK, and RK of superior temporal gyrus, and MK and AK of the hippocampus (Table 4), and the differences were significant (*p* < 0.05). There were no significant differences in AD, RD, and MD in each ROI.

**Table 4:** Correlation between peak TSB values and DKI parameters of each ROI.

		**TSB peak values**	
		***R* value**	***p*-value**
Globus Pallidus	MK	0.534	0.000^*^
	AK	0.274	0.016^*^
	RK	0.462	0.000^*^
	FA	0.026	0.420
	MD	0.172	0.096
	AD	0.022	0.886
	RD	−0.124	0.416
Thalamus	MK	0.527	0.000^*^
	AK	0.189	0.075
	RK	0.475	0.000^*^
	FA	−0.165	0.125
	MD	−0.098	0.218
	AD	0.132	0.388
	RD	0.102	0.507
Auditory Radiation	MK	0.586	0.000^*^
	AK	0.414	0.000^*^
	RK	0.456	0.000^*^
	FA	−0.080	0.261
	MD	−0.223	0.051
	AD	−0.345	0.080
	RD	0.238	0.115
Superior Temporal Gyrus	MK	0.423	0.000^*^
	AK	0.357	0.002^*^
	RK	0.334	0.004^*^
	FA	−0.081	0.272
	MD	−0.213	0.052
	AD	0.110	0.474
	RD	0.147	0.336
Hippocampus	MK	0.343	0.004^*^
	AK	0.352	0.003^*^
	RK	0.277	0.171
	FA	0.081	0.267
	MD	0.061	0.312
	AD	−0.039	0.798
	RD	0.202	0.184

*R was a correlation coefficient*.

* *p < 0.05 was considered to indicate a statistically significant difference*.

### Denver Development Screening Test

In 76 of the discharged newborns, only 40 parents (53%) completed a phone survey and completed DDST, 2 parents (2%) rejected a phone survey, and 34 parents (45%) did not answer the phone. According to the result of DDST taken by 40 parents, 3 children were placed in the suspect group, 6 children were placed in the abnormal group, and 31 children were placed in the normal group. Among the abnormal DDST children, only one child was placed in the moderate TSB group, while the other five children were placed in the severe TSB group. The abnormal group results reveal different affected areas of development: three infants (gross motor and language delay), one infant (fine motoradaptive, language and gross motor delay), and two infants (personal-social, fine motoradaptive, language and gross motor delay). In the severe group, the proportion of abnormal DDST children was statistically significantly increased compared with that of the mild and moderate groups (*p* < 0.05).

Compared with the normal group, the AK value of inferior olivary nucleus showed significant differences (*t* = 2.323, *p* < 0.05) in the suspicious abnormal group, and the MK of globus pallidus (*t* = 4.023), temporal gyrus (*t* = 3.264), and auditory radiation (*t* = 2.913); RK of globus pallidus (2.257), dorsal thalamus (*t* = 2.318), and auditory radiation (*t* = 2.212); and MD of globus pallidus (*t* = 2.431) showed significant differences (*p* < 0.05) in the abnormal group. There were significant differences with MK of temporal gyrus and auditory radiation, AK of temporal gyrus and hippocampus, and MD of globus pallidus in the case group [including the suspicious abnormal group and the abnormal group (*p* < 0.05) compared with the normal group].

## Discussion

Bilirubin-induced encephalopathy, which is caused by free plasma indirect bilirubin crossing the blood–brain barrier to the central nervous system, can be transient and reversible (7). Bilirubin encephalopathy is reported all over the world; however, more cases of the disease are currently reported in less developed countries than developed countries (20). The presence of bilirubin in the brain causes apoptosis of neurons in the basal ganglia, cerebellum, and brainstem, leading to kernicterus, which is also known as chronic bilirubin encephalopathy. Death or lifelong disabilities can be the result of kernicterus (21). In this study, DDST testing showed that the prognosis of children with bilirubin encephalopathy was related to bilirubin level to some extent, and children with higher bilirubin levels might have a relatively poor prognosis.

In regards to the difficulties of observing deposited bilirubin in the brain nuclei, diagnosis of bilirubin encephalopathy can be achieved by observing the clinical characteristics of patients, abnormal imaging on brain MRIs, and abnormal auditory brainstem responses. The globus pallidus, substantia nigra, basal ganglia, thalamic nuclei, hippocampus, dentate, inferior olives, and cerebellum are the most commonly affected areas of the brain to the toxicity induced by bilirubin (12).

MRI is an important diagnostic examination for diagnosing acute and chronic bilirubin encephalopathy. MRI images recorded during the acute phase of kernicterus have an abnormally increased signal intensity in T1-weighted imaging in the globus pallidus and subthalamic nuclei (10, 22, 23). In this study, only 48 of 76 newborns (63.2%) showed bilateral, symmetric, abnormally increased signal intensity in the globus pallidus in T1-weighted imaging. No change in signal intensity was observed in our cases in T2-weighted imaging. According to serum bilirubin, the patients with increased T1 intensity in the globus pallidus were distributed in the mild group (10 newborns), moderate group (18 newborns), and severe group (20 newborns). The high signal positive rate of globus pallidus on MRI T1WI in different groups increases with serum bilirubin and there was a significant difference between the positive rate and serum bilirubin level (*p* < 0.05). In the remaining cases, the reason why there was no increase in globus pallidus and subthalamic nuclei signals is unknown, although reasons may include stronger resistance mechanisms, or that damage to the brain was under the minimum level detected by MRI, or that abnormal signal changes monitored by MRI disappeared as a result of the death of neurons (24).

Advanced neuroimaging techniques including DTI and DKI have been applied in a wide range of studies of central nervous system (CNS) and neurodegenerative diseases (13, 25). DTI is a technology that uses the irregular free movement of water molecules to excite the EPI sequence and reflects the microstructure of nerve fibers. These findings suggest that FA may serve as an *in vivo* marker for ABE. DTI has not been used extensively to study ABE, and some research reveals decreased FA in the GP (2). During brain development, many cellular processes can affect water diffusion properties. WM myelination and maturation, axonal growth and development, and changes in axonal membrane permeability can affect free water diffusion (26). The DTI technique has been shown to be sensitive to age-related microstructural changes in both rodent and human models (27, 28). DTI considers the diffusion of water in the brain as a Gaussian distribution; hence, it is unable to detect the diffusion heterogeneity in biological tissues. Compared with DTI, DKI was proposed to describe the non-Gaussian water diffusion (restricted and hindered diffusion) behavior in neural tissues (29), and it more accurately reflects the real situation of the diffusion motion of the free water molecules in the biological tissue and better reflects the complexity and inhomogeneity of the tissue microenvironment (30). A previous rodent study ascertained that DKI offers a more sensitive evaluation of the microstructural complexity of both WM and GM at different stages of brain development compared with DTI (28), and kurtosis metrics of DKI (particularly MK) may provide additional information about brain maturation compared with that obtainable with conventional diffusion tensor metrics (specifically FA). Therefore, the DKI technique was introduced as a mathematical extension of DTI.

DKI is sensitive in detecting pathology in the GM (13) as well as in WM (14). Therefore, the ability to detect anisotropic as well as isotropic diffusion makes DKI an important diagnostic tool, supporting the possibility that DKI might become a sensitive early-stage biomarker for many CNS diseases (15–18). DKI provides not only the diffusion tensor metrics (FA, AD, and RD) but also the kurtosis metrics (AK, RK, and MK). This study tried to evaluate ABE using DKI.

Compared with the control group in this study, MK, AK, and RK of globus pallidus increased remarkably in the severe group and there was a significant positive correlation with TSB, while there were no significant differences in the mild and moderate group. The MK and RK of subthalamic nuclei increased in the moderate and severe group, showing significant differences compared with the control group. The previous study (19) observed the decreased kurtosis values in neonatal bilirubin encephalopathy, which differed from this study. The different included subjects and the timing that MRI scans taken in this study may result in the different trend of kurtosis values. The FA of globus pallidus and subthalamic nuclei showed no significant differences in all groups. This may indicate that damage of globus pallidus and subthalamic nuclei are relative to the serum bilirubin concentration, and kurtosis metrics are more sensitive to the changes of microstructure than FA. AK and RK are of interest for white matter bundles, which provide additional information regarding the axonal and myelin integrity (31). The change of AK and RK demonstrated that the water diffusion may be limited and the fiber structure of the globus pallidus and subthalamic nuclei may be affected by the cytotoxic effect of bilirubin. MK value is able to detect the changes in microstructure in both white matter and gray matter (32). MK value has shown increase with brain maturation in both GM and WM (28, 29), which is likely due to continuing myelination, increased cell packing density, and increased microstructural complexity, and the MK value increase for detection of microstructural changes in traumatic brain injury (33), Parkinson's disease (15), and grading gliomas (34, 35). A change in the MK value depends on the structural complexity of the ROIs (36, 37). Increases in GM microstructural complexity may indicate an increase in dendrites = spines, synaptic pruning = refinement, or changes in cell packing density (38). In the case group of our study, kurtosis values especially MK increased in the globus pallidus, dorsal thalamus, frontal lobe, auditory radiation, superior temporal gyrus, hippocampus, and putamen. The pathological basis for the abnormal signal intensity may be astrocyte cell reaction, bilirubin accumulation in the nerve cells, loss of neurons, demyelination, and nerve cell membrane damage by bilirubin neurotoxicity (39, 40), which could increase the complexity of GM resulting in increasing kurtosis values. In this study, kurtosis metrics provided a sensitive readout and DKI seems to be more useful in detecting ABE pathology than DTI. However, our findings suggest that DTI indexes have limited value in the diagnosis of ABE.

The auditory system is highly sensitive to unconjugated bilirubin, but the mechanism of its neuron damage is not fully understood. Previous research indicated that the incidence of auditory nervous diseases is higher with the increase of serum bilirubin concentration (41). Auditory radiation and superior temporal gyrus are the main components of the auditory transduction pathway. In this study, MK, AK, and RK of auditory radiation and RD, MK, and RK of superior temporal gyrus in the severe group increased compared with the control group. These results showed significant differences and indicated that the changes might be relative to the serum bilirubin concentration. We assumed that increased MK value may be mainly due to the astrocyte reaction caused by bilirubin toxicity, the deposition of bilirubin in nerve cells, or the destruction of nerve cell membranes by bilirubin. The DKI changes may help us to better understand fiber bundle morphology changes of the auditory transduction pathway in children with hearing impairment caused by bilirubin encephalopathy, but whether this can guide rehabilitation treatment remains to be clarified by further research.

Strengths of this study include the use of the newer MRI approach to study and stage the effect of bilirubin on the CNS, and the ability of DKI to reflect the subtle structural changes and severity of neonatal acute bilirubin encephalopathy. However, our study had three main limitations. First, the small sample size may have influenced the results. In the future, we will increase the sample size to investigate the correlation between structural alterations of different causes. Second, although we observed structural alterations in acute bilirubin encephalopathy, the structural changes in other phases of the hyperbilirubinemia in infants is not known. Third, the limited coordination of infants may affect the image quality, but improvements in the optimization of DKI technology may reduce the scanning time and improve image quality. In future studies, we will conduct regular follow-ups, monitoring, and evaluation of children so that the longitudinal dynamic changes in brain structure can be examined.

## Conclusion

This study showed that kurtosis metrics have comparable performance in the discrimination of regions of the ABE as compared to conventional diffusion metrics. DKI can reflect the subtle structural changes of neonatal acute bilirubin encephalopathy, and MK is a sensitive indicator to indicate the severity of brain damage.

## Data Availability Statement

The original contributions presented in the study are included in the article, further inquiries can be directed to the corresponding author.

## Ethics Statement

The studies involving human participants were reviewed and approved by Ethics Committee of the second Affiliated Hospital of Shantou University Medical College. The patients/participants provided their written informed consent to participate in this study.

## Author Contributions

JL and WZ designed the research. JL, HZ, and QL performed the research and analyzed the data. HZ and JL wrote the article. All authors contributed to the article and approved the submitted version.

## Conflict of Interest

The authors declare that the research was conducted in the absence of any commercial or financial relationships that could be construed as a potential conflict of interest.

## Publisher's Note

All claims expressed in this article are solely those of the authors and do not necessarily represent those of their affiliated organizations, or those of the publisher, the editors and the reviewers. Any product that may be evaluated in this article, or claim that may be made by its manufacturer, is not guaranteed or endorsed by the publisher.
